# Air Embolism into Superior Mesenteric Artery Following Replacement of Ascending Aorta for Aortic Dissection – A Rare and Fatal Case

**DOI:** 10.5334/jbsr.822

**Published:** 2018-10-18

**Authors:** Lukas Lambert, Tomas Grus, Rudolf Spunda, Martin Balik, Stanislav Trca

**Affiliations:** 1 Department of Radiology, First Faculty of Medicine of Charles University in Prague, CZ; 2 Department of Cardiovascular Surgery, First Faculty of Medicine, Charles University in Prague, CZ; 3 Department of Anesthesiology and Intensive Care, First Faculty of Medicine, Charles University in Prague, CZ; 4 Department of Surgery, First Faculty of Medicine, Charles University in Prague, CZ

**Keywords:** mesenteric ischemia, aortic dissection, air embolism

## Abstract

We report an unusual case of fatal air embolism into the superior mesenteric artery in a patient, who underwent replacement of the ascending aorta for aortic dissection type A. CT performed twice on the first postoperative day showed abundant air in the superior mesenteric artery and its branches (but not in the portal-venous system) indicating air embolism with no signs of bowel necrosis. On the second postoperative day, the patient underwent extensive bowel resection due to bowel ischemia and died on the third postoperative day on MODS/SIRS.

## Case Report

**Figure 1:**
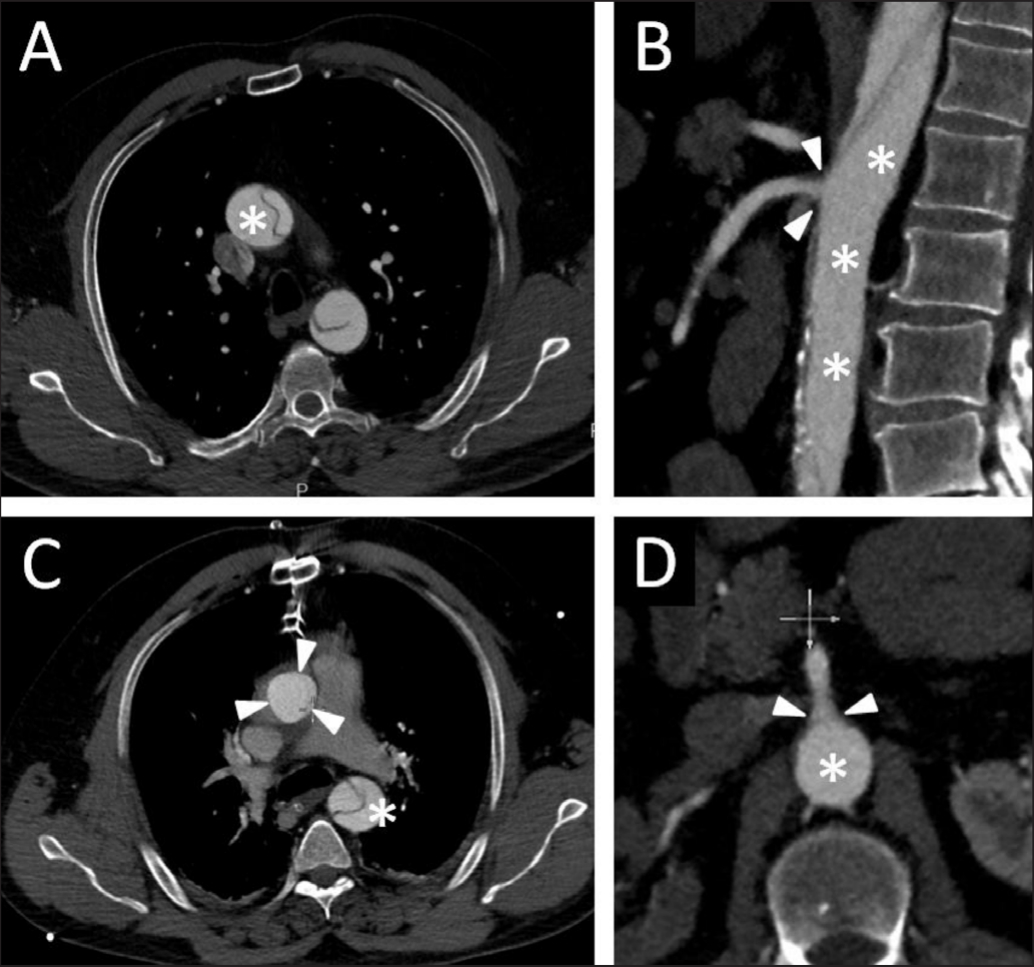
CT angiography of the aorta shows aortic dissection before **(a)** and after repair **(b)**. The ostium of the superior mesenteric artery (**c, d**, arrowheads) is supplied from the false lumen (asterisks).

A 50-year-old male patient with aortic dissection originating just above the aortic valve and extending down to the common iliac arteries (Stanford A, Figure 1) underwent immediate surgery with repair of the ascending aorta in moderate hypothermia under cardiopulmonary bypass. The arterial line was inserted in the right axillary artery, the vent was placed in the right upper pulmonary vein, and two-stage venous line was inserted through the right auricula. The intact aortic valve was resuspended. Extracorporeal circulation was suspended after induction of moderate hypothermia (25°C) and the aortic cross-clamp from the ascending aorta was removed. The false lumen was then glued and an open distal anastomosis to a prosthetic graft was constructed. Then, extracorporeal circulation was resumed, systemic circulation was deaired and the patient was warmed.

**Figure 2:**
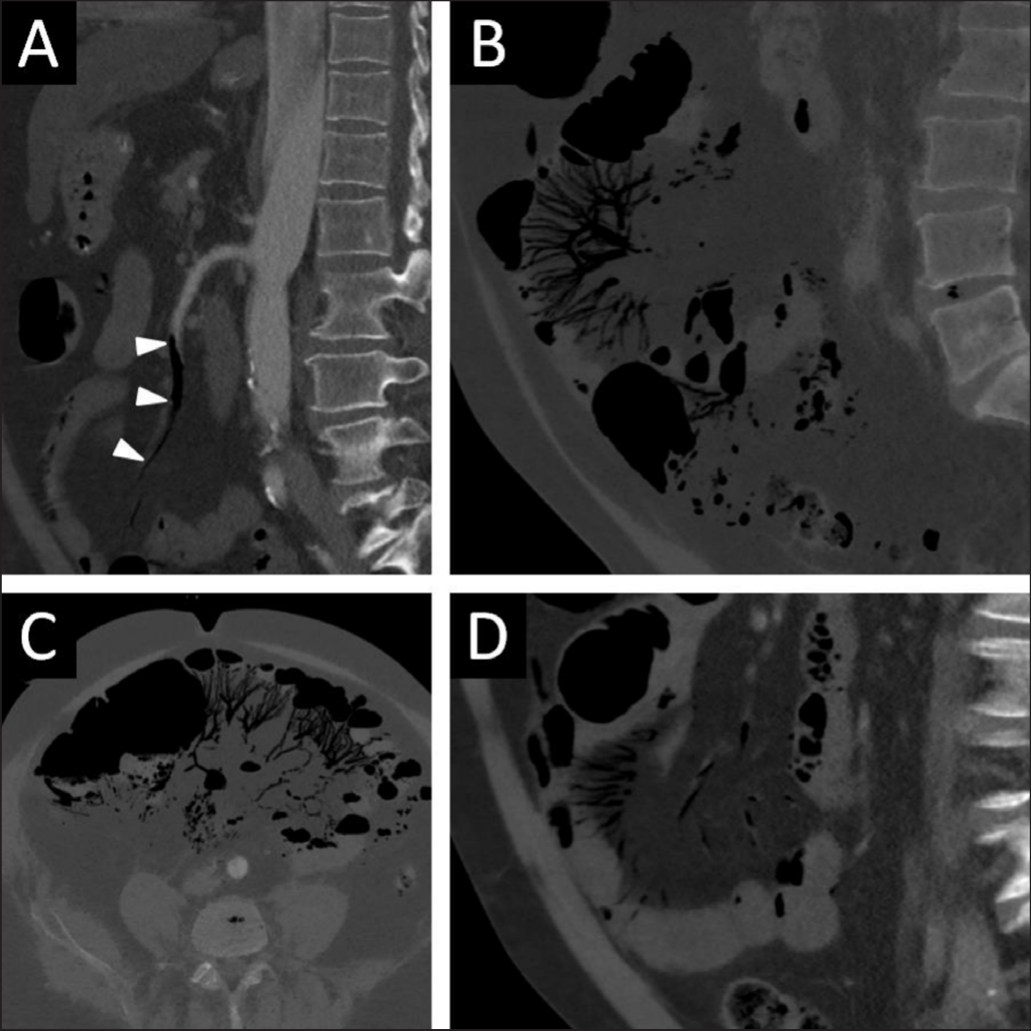
Gas bubbles in the superior mesenteric artery **(a)** and its branches extending up to the arcades **(b)** shown on the early CT on the first postoperative day. Small bowel loops remain without significant distension and contain only a small amount of fluid. A second CT on the first postoperative day **(c, d)** 14 hours later shows that the gas bubbles migrated further into the periphery of the branches of the superior mesenteric artery and into the bowel wall.

Early on the first postoperative day, CT of the aorta was requested because of marked elevation of lactate-dehydrogenase (129 μkat/l) and signs of acute renal insufficiency (creatinine 292 μmol/l). CT showed good postoperative result in the ascending aorta, but large amount of air in the branches of the superior mesenteric artery up to the arcades was found (Figure 2). There was absolutely no air in the portal-venous system including the liver. The bowel loops were not distended, and there were no signs of bowel paralysis.

At midnight on the first postoperative day, a biphasic CT scan of the abdomen was requested due to elevated intra-abdominal pressure (18 mmHg). The CT showed distribution of the intra-arterial gas more into the periphery and into the wall of the bowel loops that still did not display signs of ileus (Figure 2). The next morning, surgical exploration was performed due to increasing intra-abdominal pressure as a sign of imminent abdominal compartment syndrome. An extensive bowel resection from oral ileum down to the splenic flexure had to be performed due to extensive necrosis of the bowel. Shortly after the operation, the patient became hypotensive with signs of overwhelming vasoparalysis and died on the third postoperative day of multiple organ dysfunction syndrome (MODS) with systemic inflammatory response syndrome (SIRS).

## Discussion

Vascular air embolism, however rare this event is, can be potentially fatal [1]. It is associated with surgery, invasive procedures (e.g. placement of central venous line), and trauma. Typical cases result in impairment of the cardiovascular, pulmonary, and central nervous systems [2]. Specifically in cardiac bypass procedures, massive air embolism is a very rare event but with adverse outcomes in 50% [1]. Pathophysiologically, the presence of air bubbles in an artery acts as an obstruction and results in acute hypoperfusion, which is proportional to the size of the bubbles, and in an inflammatory response of the endothelium with increased microvascular permeability [3,4]. When revision of the aortic arch is needed, the blood from the lumen has to be suctioned away (during suspended extracorporeal circulation). By the end of the procedure, the aorta is refilled with blood. It is very likely that some amount of air remained in the false lumen that was closed by the distal anastomosis and it became subsequently dislodged into the circulation. In a typical case, the air bubbles would predominantly enter the carotid arteries and result in cerebral ischemia. However, iatrogenic introduction of air into the false lumen of the dissection could physically bypass the branches of the aortic arch channeling the air into the visceral region. In the supine position, air bubbles accumulate in a non-dependent position, where visceral arteries originate. Interestingly, because the celiac artery and the inferior mesenteric artery were covered by the true lumen, the bubbles entered the SMA only.

Air embolism into the SMA is a very rare event that results in mesenteric hypoperfusion. Signs of bowel paralysis and gas in the portal vein may be absent due to residual perfusion of the bowel that may be grossly insufficient. Unfortunately, because of extensive distribution of gas in the mesenteric arteries up to the arcades and later in the bowel wall, endovascular aspiration cannot be expected to yield any results.
